# Human biodistribution and radiation dosimetry of the 5-HT_2A_ receptor agonist Cimbi-36 labeled with carbon-11 in two positions

**DOI:** 10.1186/s13550-019-0527-4

**Published:** 2019-07-31

**Authors:** Annette Johansen, Søren Holm, Bente Dall, Sune Keller, Jesper L. Kristensen, Gitte M. Knudsen, Hanne D. Hansen

**Affiliations:** 1 Neurobiology Research Unit and Center for Integrated Molecular Brain Imaging, Rigshospitalet, Building 6931, Blegdamsvej 9, DK-2100 Copenhagen, Denmark; 2 Faculty of Health and Medical Sciences, University of Copenhagen, Copenhagen, Denmark; 3 PET and Cyclotron Unit, Rigshospitalet, Copenhagen, Denmark; 4 Department of Drug Design and Pharmacology, Faculty of Health and Medical Sciences, University of Copenhagen, Copenhagen, Denmark

**Keywords:** ^11^C-Cimbi-36, 25B-NBOMe, 5-HT_2A_ receptor, Biodistribution, Pharmacology, Pharmacokinetics, Positron emission tomography, Radiation dosimetry

## Abstract

**Background:**

Cimbi-36 can be ^11^C-labeled to form an agonist radioligand used for positron emission tomography (PET) imaging of the 5-HT_2A_ receptor in the brain. In its non-labeled form (25B-NBOMe), it is used as a recreational drug that can lead to severe adverse effects, in some cases, with fatal outcome. We investigated human biodistribution and radiation dosimetry of the radioligand with two different radiolabeling positions. Seven healthy volunteers underwent dynamic 120-min whole-body PET scans (injection of 581 ± 16 MBq, *n* = 5 for ^11^C-Cimbi-36; 593 ± 14 MBq, *n* = 2 for ^11^C-Cimbi-36_5). Time-integrated activity coefficients (TIACs) from time-activity curves (TACs) of selected organs were used as input into the OLINDA/EXM software to obtain dosimetry information for both ^11^C-labeling positions of Cimbi-36.

**Results:**

The effective dose was only slightly higher for ^11^C-Cimbi-36 (5.5 μSv/MBq) than for ^11^C-Cimbi-36_5 (5.3 μSv/MBq). Standard uptake value (SUV) curves showed higher uptake of ^11^C-Cimbi-36 in the pancreas, small intestines, liver, kidney, gallbladder, and urinary bladder compared with ^11^C-Cimbi-36_5, reflecting differences in radiometabolism for the two radioligands. Variability in uptake in excretory organs for ^11^C-Cimbi-36 points to inter-individual differences with regard to metabolic rate and route. Surprisingly, moderate uptake was found in brown adipose tissue (BAT) in four subjects, possibly representing specific 5-HT_2A/2C_ receptor binding.

**Conclusion:**

The low effective dose of 5.5 μSv/MBq allows for the injection of up to 1.8 GBq for healthy volunteers per study (equivalent to 3 scans if injecting 600 MBq) and still stay below the international guidelines of 10 mSv, making ^11^C-Cimbi-36 eligible for studies involving a series of PET scans in a single subject. The biodistribution of Cimbi-36 (and its metabolites) may also help to shed light on the toxic effects of 25B-NBOMe when used in pharmacological doses in recreational settings.

**Electronic supplementary material:**

The online version of this article (10.1186/s13550-019-0527-4) contains supplementary material, which is available to authorized users.

## Introduction

Cimbi-36 can be labeled with carbon-11 to form a positron emission tomography (PET) radioligand for imaging of serotonin 2A receptor (5-HT_2A_R) agonist binding in the human brain [1]. 5-HT_2A_Rs are widely distributed in the cerebral cortex [2] and has been linked to neuropsychiatric disorders such as depression [3, 4]. Further, many antipsychotic medications act as antagonist or inverse agonists on the 5-HT_2A_R [5], while psychedelic effects are shown to correlate with 5-HT_2A_R occupancy following administration of the classical hallucinogen, psilocybin [6]. The Cimbi-36 molecule (*N*-(2-methoxybenzyl)-2,5-dimethoxy-4-bromophenethylamine) itself acts as an agonist on the 5-HT_2A_R and is used in pharmacological doses as a recreational drug, known as 25B-NBOMe, belonging to the class of new psychoactive substances [7].

^11^C-labeled Cimbi-36 is presently the only agonist radioligand available for imaging the 5-HT_2A_R [8], and it is known that agonists and antagonists interact in different modes with the receptor [9]. Cimbi-36 has been widely characterized in terms of in vitro receptor binding [10], preclinical evaluation of safety, pharmacological effects, and functionality as a PET tracer [11, 12]. As of yet, no studies on human biodistribution or radiation dosimetry of ^11^C-labeled Cimbi-36 have been conducted. Extrapolations from pig and rat dosimetry are available [11], but differences across species cannot be predicted [13].

Metabolism of Cimbi-36 has been of interest, not just because of its use in neuroreceptor imaging, but also due to illicit use as a recreational drug [14]. This, in turn, has shed light on the relationship between the ^11^C-labeling position and radiolabeled metabolites and their effects on binding estimates [15]. In this study, we not only compare human dosimetry estimates of two ^11^C-labeling positions of the PET radioligand Cimbi-36 (^11^C-Cimbi-36 (N-(2[^11^C-OCH_3_]methoxybenzyl)-2,5-dimethoxy-4-bromophenethylamine) vs ^11^C-Cimbi-36_5 (N-(2-methoxybenzyl)-2-methoxy-5-[^11^C-OCH_3_]-methoxy-4-bromophenethylamine) but also provide new information on biodistribution and pharmacokinetics of the controlled substance, 25B-NBOMe.

## Materials and methods

### Study design

The study was approved by the Ethics Committee for the Capital Region of Denmark (protocol no. H-15001910) and the Danish Health and Medicine Authority (EudraCT no. 2015-004256-21). The study was registered as a clinical trial (NCT02629003) at ClinicalTrial.gov and was performed in accordance with the recommendation for Good Clinical Practice. Study participants were recruited through online advertisement and all participants gave written informed consent after a detailed explanation of the study. Whole-body distribution and radiation dosimetry of ^11^C-Cimbi-36 and ^11^C-Cimbi-36_5 were investigated with PET/CT imaging. Eight healthy volunteers (mean age 20.6 ± 2.8 years, 5 females) were included in the study. The radiotracers were produced as previously described [14].

### Whole-body PET/CT acquisition

Scanning was performed with a Siemens Biograph mCT PET/CT system that was normalized daily using a Ge-68 cylinder phantom and cross-calibrated to gamma counter and radionuclide calibrator in a biweekly routine process using F-18. Prior to intravenous injection of the radiotracer (aimed at 600 MBq) in the antecubital vein, the subjects underwent a low-dose CT scan for subsequent anatomical localization. The 120-min PET scan started at the time of injection with an initial 3-min list mode acquisition over the heart (not presented here), followed by (up to) 16 whole-body scan passes with increasing scan time. Each scan pass covered a total of 198 cm in 15 bed positions (axial field of view 21.6 cm, overlap 9.0 cm). For the first 4 passes, scan time per bed position was 15 s (5 s over the legs). This was increased for pass 5–8 to 30 s (10 s) and for the final 8 passes to 60 s (20 s). PET reconstruction was done using 3 iterations and 21 subsets OSEM3D with TOF and Siemens mCT standard scatter correction, random correction, and low-dose CT-based attenuation correction. A 5-mm FWHM Gaussian post-reconstruction filter was applied.

Venous blood was drawn from the opposite antecubital vein for whole blood (WB) radioactivity measurement.

### Estimation of whole-body distribution and radiation dose

For each subject, scans were analyzed using Mirada RTx (Mirada Medical, Oxford, UK) as previously described [16, 17], but with some modifications, for the following organs: thyroid, heart wall, liver, spleen, kidney, bone, bone marrow (the central part of the L4-L5 vertebrae was used as a surrogate), stomach wall and contents, subcutaneous fat, and periclavicular brown adipose tissue (BAT), 2–3 VOIs (volume ranging from 7 mL (thyroid gland) to 63 mL (liver)) were drawn based on the PET image and cross-checked with the anatomical CT image. For the following organs, a single VOI was placed encompassing most of the organ based on the CT image and also guided by PET images; brain, lungs, gallbladder, pancreas, proximal part of the small intestines, and large intestines. To account for the change in volume of the urinary bladder during the scan, the bladder (contents) region was drawn based on the PET images for subjects scanned with ^11^C-Cimbi-36. This was not possible for images of ^11^C-Cimbi-36_5, as the activity could not be discerned from surrounding tissue. For each time point, decay-uncorrected activity concentration (kBq/mL) and standard uptake values (SUVs; g/mL, by definition decay-corrected) were extracted. Cumulated activity for each organ was estimated as the area under the curve (AUC; kBq/mL × h (kBq per mL times h)) for each subject using the trapezoid method. Although of minor importance since the integration already covers six half-lives of the tracer, extrapolation from the last time point to infinity was done by adding *A*_120 min_/*k (k* being the decay constant for carbon-11), thus assuming simple physical decay. These values were subsequently normalized by dividing by injected activity and then multiplied by standard organ masses of the OLINDA male or female adult phantom [18], thereby estimating the time-integrated activity coefficients (TIAC, formerly known as *residence time*; unit *h*) for each organ. Individual TIACs were scaled by the ratio of the individual subject’s body mass to standard phantom body mass, then averaged across subjects (*n* = 5 or *n* = 2) and entered into the OLINDA/EXM 2.0 software to obtain estimates of absorbed and effective doses with tissue weighting factors according to ICRP 103 [19]. With this method, we use the principle for extrapolation from animal data (known as the % injected dose/g method) [20] to human data, except that we extrapolate from a small sample of humans to the general population.

Urinary bladder contents data were incorporated using the bladder voiding model in OLINDA as a practical way to estimate absorbed dose to the bladder wall. For each subject scanned with ^11^C-Cimbi-36, cumulated decay-corrected activity (in kBq) was plotted over time and fitted using a one phase association equation in GraphPad Prism (GraphPad Prism version 8.0.0 for MacOS, GraphPad Software, San Diego, CA, USA, www.graphpad.com). The results of these fits were inserted into the Olinda software, and the returned urinary TIACs were averaged across subjects. A bladder voiding interval of 2 h was used, which essentially means that activity is accumulated and excreted only once, since less than 2% of the activity is left after 2 h. TIACs of remainder tissue were calculated as the total number of decays minus the sum of the organ-specific values. For ^11^C-Cimbi-36_5 we used post-scan cumulated urine activity to yield excretory fraction estimate.

## Results

### Dosimetry estimation

Five participants (three females, two males) completed ^11^C-Cimbi-36 PET/CT scans (injection of 581 ± 16 MBq; specific activity at the time of injection was 665 ± 240 GBq/μmole; 0.37 ± 0.15 μg) according to protocol. Two participants (two females) completed the ^11^C-Cimbi-36_5 PET/CT scan (injection of 583–603 MBq; specific activity at the time of injection was 365–583 GBq/μmole; 0.38–0.63 μg) according to protocol. We originally planned to include 10 subjects, but 1 female participant did not complete the scan as the production of radiotracer failed. We refrained from completing the last two planned ^11^C-Cimbi-36_5 scans, as the decision to use the ^11^C-Cimbi-36 labeling position for future 5-HT_2A_R imaging studies was made before the completion of this study [15], regardless of the dosimetry outcome. Thus, for ethical reasons the study was halted. No adverse events occurred.

**Fig. 1:**
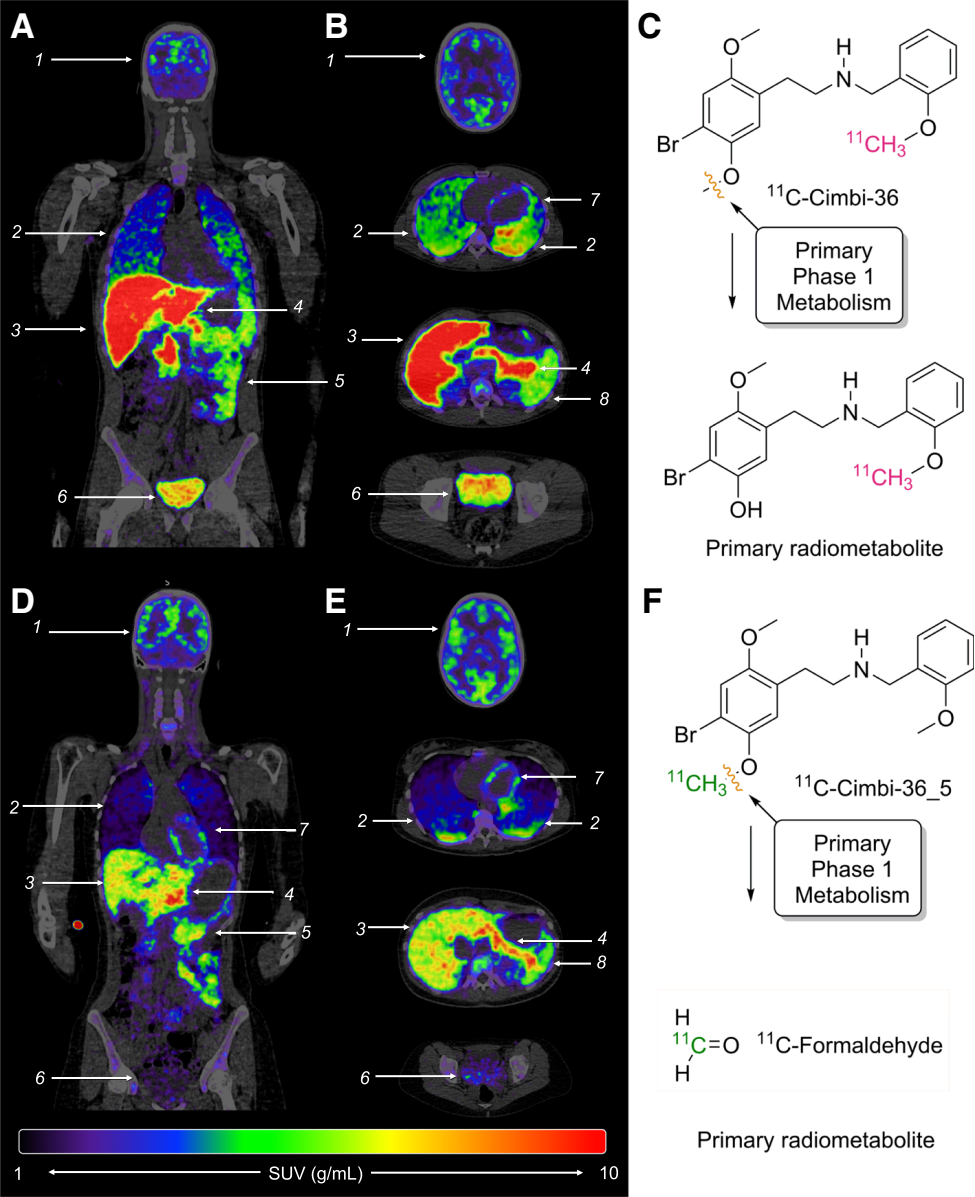
Coronal and horizontal PET/CT fused images of ^11^C-Cimbi-36 (**a**, **b**) and ^11^C-Cimbi-36_5 (**d**, **e**) 40 min into the scan. Brain (1), lungs (2), liver (3), pancreas (4), small intestines (5), urinary bladder (6), heart wall (7), spleen (8). Primary phase 1 metabolic route resulting in different radiometabolites for ^11^C-Cimbi-36 (**c**) and ^11^C-Cimbi-36_5 (**f**)

Whole-body PET/CT images 40 min into the scan are shown for both radioligands in Fig. 1. Data from the organ VOIs (see Additional file 1: Supplementary Table S1 for the individual TIACs) were quantified and processed using the Olinda software, yielding absorbed and effective doses, as seen in Table 1.

**Table 1:** Organ absorbed doses and contributions to effective doses for ^11^C-Cimbi-36 and ^11^C-Cimbi-36_5

	Absorbed dose (μGy/MBq)		Contributions to effective dose (μSv/MBq)	
Target organ	^11^C-Cimbi-36	^11^C-Cimbi-36_5	^11^C-Cimbi-36	^11^C-Cimbi-36_5
Adrenals	5.83	5.52	0.05	0.05
Brain	6.12	6.72	0.06	0.07
Breasts	2.38	2.42	0.29	0.29
Esophagus	3.54	3.50	0.14	0.14
Eyes	2.19	2.32	0.00	0.00
Gallbladder wall	5.85	4.88	0.05	0.05
Left colon	5.24	5.70	0.25	0.28
Small intestine	5.55	4.91	0.05	0.05
Stomach wall	5.46	7.56	0.66	0.91
Right colon	3.23	3.15	0.16	0.15
Rectum	2.99	2.57	0.07	0.06
Heart wall	10.70	9.84	0.10	0.09
Kidneys	11.80	10.50	0.11	0.10
Liver	15.70	13.20	0.63	0.53
Lungs	8.61	8.31	1.03	1.00
Ovaries	3.14	2.91	0.13	0.12
Pancreas	13.30	11.60	0.12	0.11
Prostate	2.67	2.29	0.01	0.01
Salivary glands	2.36	2.48	0.02	0.02
Red marrow	4.19	4.33	0.50	0.52
Osteogenic cells	3.37	3.72	0.03	0.04
Spleen	18.40	17.90	0.17	0.17
Testes	1.89	1.87	0.08	0.07
Thymus	3.20	3.21	0.03	0.03
Thyroid	6.37	6.52	0.26	0.26
Urinary bladder wall	12.40	2.40	0.50	0.10
Uterus	3.51	2.85	0.02	0.01
Effective dose			5.5	5.3

The effective doses were 5.5 μSv/MBq and 5.3 for ^11^C-Cimbi-36 and ^11^C-Cimbi-36_5, respectively. For ^11^C-Cimbi-36, the organs with the highest contributions to effective dose were the lungs, urinary bladder, liver, stomach wall, and red marrow, all contributing more than 0.50 μSv/MBq. For most organs, ^11^C-Cimbi-36_5 yielded lower absorbed doses, with the highest contribution to effective dose (> 0.50 μSv/MBq) found again in the lungs, stomach wall, liver, and red marrow. The most noticeable difference between the radioligands was found in the urinary bladder (ratio of 5).

No discernible radioactivity uptake was seen in the reproductive organs (uterus, ovaries, testes) for ^11^C-Cimbi-36 (three females, two males) or ^11^C-Cimbi-36_5 (two females). Thus, the absorbed and effective doses are based on radiation from neighboring organs and the remainder activity. In the case of ^11^C-Cimbi-36_5, radiation to the testes is based on extrapolation to the male phantom in Olinda (Table 1).

### Biodistribution

**Fig. 2:**
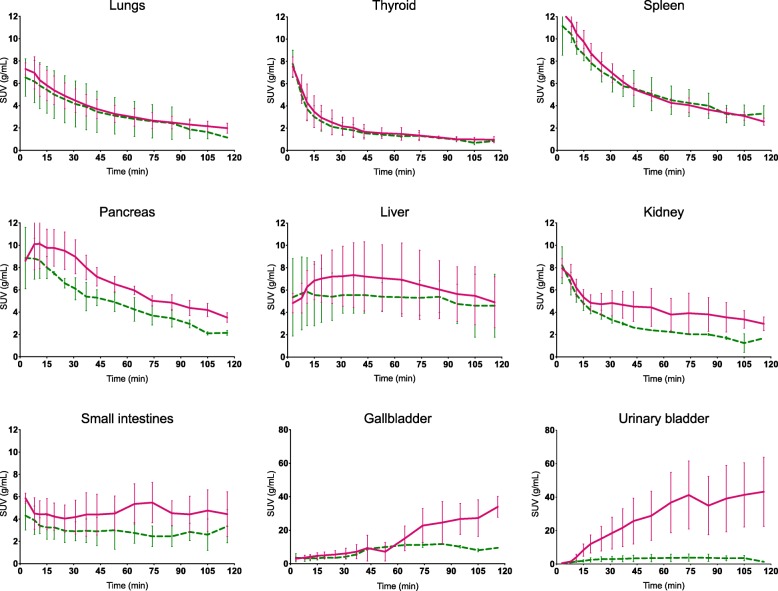
Standard uptake value (SUV) curves for selected organ VOIs. Full line (pink) denotes ^11^C-Cimbi-36 and dotted line (green) denotes ^11^C-Cimbi-36_5. Note the different scale of SUV curves for the gallbladder and urinary bladder. Error bars represent standard deviation

**Fig. 3:**
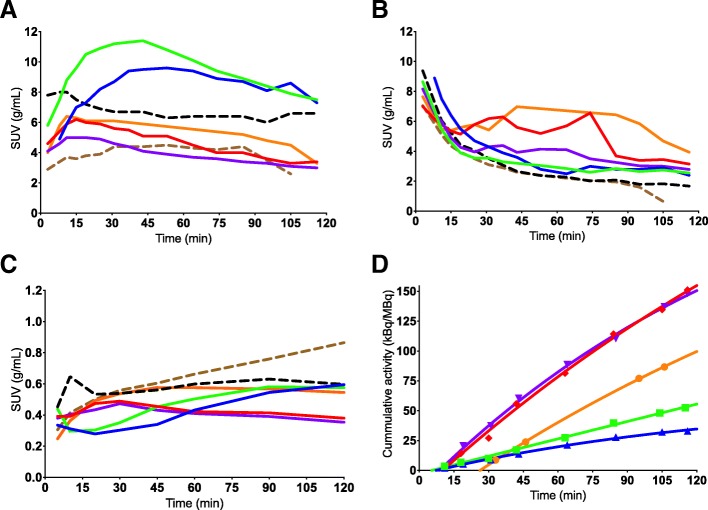
Standard uptake value (SUV) curves for individual subjects for ^11^C-Cimbi-36 (full lines) and ^11^C-Cimbi-36_5 (dotted lines) in the liver (**a**), kidney (**b**) and whole blood (**c**). Decay-corrected cumulated activity (normalized to injected activity) in the bladder fitted with monoexponential function for ^11^C-Cimbi-36 shows great variability in urinary excretion (**d**). Each color represents a single subject

SUV curves for both radiotracers (including their respective radiolabeled metabolites) are shown in Fig. 2 for selected organs, and individual subjects’ SUV curves for whole blood and excretory organs in Fig. 3. For the lungs, thyroid, and spleen, the curves are virtually identical for the two radioligand-labeling positions, with the spleen showing the highest initial uptake (> 12 g/mL). The pancreas, small intestines, liver, kidney, gallbladder, and urinary bladder all have a higher uptake of ^11^C-Cimbi-36 compared with ^11^C-Cimbi-36_5. This difference emerges after varying time intervals for the organs; liver (app. 15 min), kidney (20 min), gallbladder (60 min), and urinary bladder (10 min), and for the gallbladder and urinary bladder, it continues to increase throughout the scan.

**Fig. 4:**
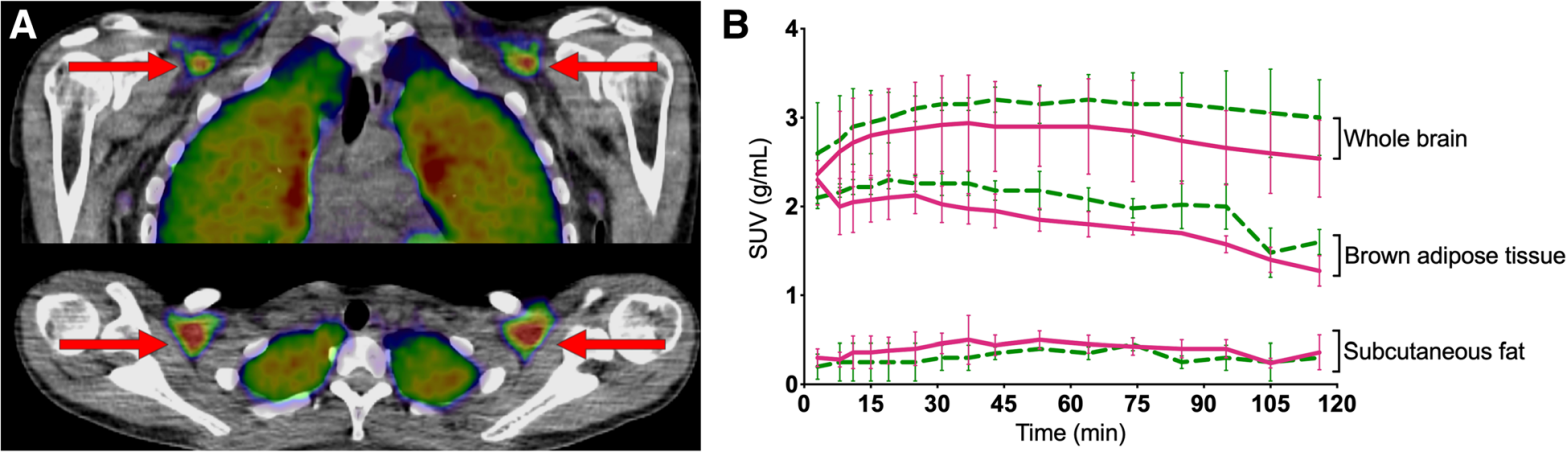
Anatomical localization of uptake in presumed brown adipose tissue (BAT) (**a**). Standard uptake value (SUV) curves for BAT, whole brain, and subcutaneous fat (**b**). Full line (pink) denotes ^11^C-Cimbi-36 and dotted line (green) denotes ^11^C-Cimbi-36_5

Moderate uptake in four of the seven participants was, surprisingly, found in what we believe to be brown adipose tissue (Fig. 4a). Localized on the side of the neck and close to the clavicular bone, we found fatty tissue (verified through visual inspection [21] and corroborated by Hounsfield unit values) with clearly detectable uptake. The subjects with these findings were all female (mean age 23 years, range 19–25; mean body mass index 21.9, range 18.8–27.9), two had been scanned with ^11^C-Cimbi-36 and two with ^11^C-Cimbi-36_5. The SUV curves in BAT closely resembles the whole brain SUVs, and in order to distinguish the uptake from non-specific binding to fatty tissue in general, we compared them with SUVs derived from a VOI in subcutaneous fat of the abdomen (Fig. 4b).

## Discussion

### Dosimetry estimates

In this study, we evaluated human radiation dosimetry for the ^11^C-labeled 5-HT_2A_R agonist PET radioligand, Cimbi-36, using two different ^11^C-labeling positions. Effective dose for ^11^C-Cimbi-36, the labeling position of choice for neuroimaging [15], was 5.5 μSv/MBq, resulting in a radiation dose of 3.3 mSv for a PET scan following injection of 600 MBq and allows for injection of 1.8 GBq per study in order to stay below 10 mSv, which is the recommended limit for studies involving healthy volunteers, that provide *intermediate* to *moderate* benefits to society [22].

^11^C-Cimbi-36 is a radioligand developed for brain imaging, and thus magnetic resonance imaging is often used for anatomical purpose, yielding no additional radioactive exposure. However, a low-dose brain-only CT for attenuation correction can be performed at a fraction of the PET effective dose.

Because of the short half-life of carbon-11 (20.4 min), organs with high perfusion tend to get the highest absorbed doses of radiation from ^11^C-labeled radioligands, but also excretory organs such as the liver, kidney, gallbladder, and urinary bladder get high exposure [23]. Indeed, this is also true for ^11^C-Cimbi-36, as the liver, kidneys, and urinary bladder are among the five organs with highest absorbed doses. This reflects the relatively fast metabolism seen for Cimbi-36 [1, 15].

The effective dose for the alternative labeling position, ^11^C-Cimbi-36_5, was only slightly lower; 5.3 μSv/MBq (equivalent to a radiation dose of 3.2 mSv if injecting 600 MBq), with the difference being most pronounced for the urinary bladder, i.e., a fivefold reduction in absorbed dose. Despite the small sample size, the generally lower uptake for ^11^C-Cimbi-36_5, including in the urinary bladder, can be attributed to the higher level of radioactivity in the form of small diffusible substances (^11^C-formaldehyde, ^11^C-formic acid), and possibly also volatile (^11^C-CO_2_) substances, comprised in the M1 radiometabolite fraction (Fig. 1) [15].

The difference in effective dose between the two ^11^C-labelings of Cimbi-36 is within the limitations of the method and inter-individual variability. Thus, even the relatively fast metabolism of parent tracers (resulting in radiometabolites with different physical properties) does not affect dosimetry outcomes noticeably. This attests to the notion that dosimetry of ^11^C-labeled radioligands is mostly dependent on the initial blood perfusion phase.

The effective doses of both radioligands are in line with other ^11^C-labeled PET tracers; range 3.0–7.8 μSv/MBq, with the exception of one (^11^C-WAY-100635; 14.1 μSv/MBq) [13, 23]. The estimated effective dose for ^11^C-Cimbi-36 in this study is also in accordance with preclinical studies; effective dose was found to be 4.9 μSv/MBq and 7.7 μSv/MBq, when extrapolating from pig and rat dosimetry, respectively [11]. In the case of ^11^C-Cimbi-36, studies in pigs thus proved to have a better translational value compared with rats, but differences across species cannot be predicted [13]. The decision of whether to undertake human radiation dosimetry studies of a new radioligand, when ^11^C-labeled tracers show this limited variability, should therefore be considered. Dosimetry studies are both costly and time-consuming, and expose healthy individuals to radiation, with (perhaps) no added benefit compared with a conservative estimate based on the highest reported effective dose, as suggested in one study [13]. A generic model for ^11^C-labeled substances for brain imaging predicts an effective dose of 4.5 μSv/MBq, based on the assumption that radioactivity is rapidly and uniformly distributed throughout all tissue [24]. Yet, in the absence of preclinical data or model that can predict with sufficient certainty if one organ receives an excessive absorbed dose, it can be justified to conduct a small number of whole-body scans to take this possible scenario into account.

Despite similar radiation dosimetry for the two tracers, ^11^C-Cimbi-36 continues to be the preferred radioligand for 5-HT_2A_R neuroimaging studies, as it has a better signal-to-noise ratio in the brain [15].

### Biodistribution, pharmacology, and metabolism

Biodistribution can help shed light on pharmacokinetics [25], but interpretation of the SUV curves for the internal organs should be done with caution, as they represent both the parent tracers and their radiolabeled metabolites. This leaves the following possible interpretations of radioactivity uptake: 1) specific binding of parent tracer; 2) enzymes or other sites of metabolism of parent tracer or labeled metabolites; 3) excretion of parent tracer or radiometabolites; 4) non-specific binding of parent tracer or radiometabolites. As biodistribution of Cimbi-36 is particularly interesting because of its use as a recreational drug (alias 25B-NBOMe) with many case reports of toxicity and even fatalities [26], we will discuss 1–3 in more detail in the following.

Specific binding: Cimbi-36 not only binds to the 5-HT_2A_R, but also has affinity for, e.g., the 5-HT_2C_ receptor and Sigma 2 receptors; however, the affinity for these two receptors are 15- [27] and 120-fold [10] lower, respectively. As no blocking agent was used in the present study, potential 5-HT_2A/C_R binding in extracerebral tissue cannot be determined with complete certainty from our data. The primary focus of 5-HT is on its effects in the central nervous system, but peripheral 5-HT is implicated in many bodily functions, such as energy metabolism through actions in the gut, pancreas, and fatty tissues [28–31] but also immune response, inflammation [32], and pain stimuli [33]. High uptake in, e.g., the small intestines or the pancreas might thus represent specific 5-HT_2A/2C_R binding. 5-HT_2A_Rs are also found on human alpha, beta, and delta cells in the pancreas [29]. In type 2 diabetic patients, increased expression of the 5-HT_2A_R was found in unspecified pancreatic islet cells relative to healthy controls [34], while 5-HT_2C_R inhibits insulin secretion by beta cells in a diabetic mouse model [35]. These findings may in part explain the hyperglycemia seen in several cases of NBOMe intoxication [7].

Surprisingly, we found high uptake in what we believe to be brown adipose tissue (BAT). A newly conducted study links administration of 25B-NBOMe in rats to hyperthermia and thermogenesis of BAT [36]. These effects are thought to be mediated through peripheral 5-HT_2A_Rs as central adrenergic and serotonergic neurons were selectively destroyed by neurotoxins in these animals. BAT stimulation by 5-HT has been shown to inhibit beta-adrenergic signaling and BAT thermogenesis [29] through the 5-HT_3_R [30, 37], while 5-HT_2A_R stimulation increases BAT thermogenesis [38, 39]. Thus, peripheral 5-HT might have bidirectional physiological effects on BAT thermogenesis, depending on 5-HT receptor expression pattern, which in turn could reflect differences in the physiological state (active vs. inactivated BAT). Interestingly, hyperthermia is a common complication in NBOMe intoxications, with and without seizures [7], and peripheral 5-HT_2A/2C_R effects on BAT might be a contributing factor.

The severe toxicity of 25B-NBOMe and other 5-HT_2_R selective agonists is curious in light of the relatively low toxicity of classical non-selective 5-HT_2A_R hallucinogens such as psilocybin, LSD, and mescaline [40, 41]. Considering the different effects on BAT thermogenesis mediated by different 5-HT receptors, toxicity might then arise because of, rather than despite, high selectivity. Other factors, such as inter-individual differences in metabolism might also contribute, as discussed below.

Metabolism: Not surprisingly, the liver showed high uptake beyond the initial perfusion phase reflecting the extensive metabolism of the parent tracers. In both pig and human, the metabolic route is through *O*-demethylation (phase I reaction, Fig. 1), primarily at the 5’-position, followed by glucuronide conjugation (phase II reaction) [14]. ^11^C-labeling in the 2-methoxybenzyl-position (^11^C-Cimbi-36) gives rise to two radiometabolite fractions: M1, comprising small polar radiometabolites, which is likely a mixture of ^11^C-formaldehyde, ^11^C-formic acid, and ^11^C-CO_2_/bicarbonate; and M2, which was identified as a ^11^C-glucuronide conjugate [14]. Changing the ^11^C-labeling to the 5′-methoxy-4-bromophenethylamine position (^11^C-Cimbi-36_5) eliminates the radiolabeled form of the glucuronide conjugate (M2), leaving only the M1 fraction [15]. Caspar et al. [42] found that the *O*-demethylation is catalyzed by CYP2C19 (cytochrome P-450 enzyme) and CYP2D6, and the relative contribution to hepatic clearance was estimated to be 69% for CYP2D6, with the remaining clearance attributed to CYP2C19 and CYP3A4 (catalyzing *N*-dealkylation and/or hydroxylation). These CYP isoforms are also found in the small intestines [43], which we hypothesize account for part of the high uptake of ^11^C-Cimbi-36 in the proximal small intestine throughout the scan. The other part possibly representing specific binding to 5-HT_2A/2C_Rs. ^11^C-Cimbi-36 also shows higher uptake than ^11^C-Cimbi-36_5 in the liver, which we speculate is because the resulting phase I metabolite of the former goes on to being glucuronidated by glucuronosyltransferases (UGT) [44]. The labeled metabolites of ^11^C-Cimbi-36_5 are small polar, likely volatile, substances [15] and therefore not likely retained in the liver to the same degree. The same pattern is seen in the kidneys, in which several UGTs are present [43], although transporters in the renal tubule cells responsible for the secretion of the ^11^C-labeled glucuronide conjugate might better explain the difference in uptake seen between the tracers. This is further substantiated as the difference in uptake does not emerge until approximately 20 min into the scan, when most of the activity is in the form of the glucuronide conjugate [15].

Inter-individual differences due to genetic variability in CYP isoforms or induction/inhibition by foods or drugs may contribute to the toxicity of NBOMes. We note that the red and purple lines are near identical in whole blood (Fig. 3) and follow the same shape in the liver, kidney, and bladder. The case is similar for the blue and green lines, which follow a different shape and have higher uptake in the liver, while the orange is somewhat in-between. The liver transit time may thus reflect differences in CYP profiles with regard to metabolic rate or route.

Excretion: The two organs with the most pronounced difference in uptake between the two labeling positions were the urinary bladder and the gallbladder, pointing to an unambiguous difference in excretory pathways of the ^11^C-labeled metabolites of the two tracers. For the gallbladder, this difference is not reflected in the dosimetry results, as the onset of the difference happens after three half-lives of the radioactive label. It is important to keep in mind that, unlike most of the organs in the human body, the urinary bladder and the gallbladder do not have fixed volumes but expand and contract on physiological demand, and thus the concentrations depend on two independent variables. Emptying by the gallbladder into the small intestines might also contribute to the high uptake in the proximal small intestines, but then again, these SUVs are relatively stable during the course of the scan. For ^11^C-Cimbi-36, the urinary bladder SUV curves (Fig. 2) vary immensely between the subjects as was the case for the absolute amount of excreted substance (Fig. 3d). As the cold doses in our study (ranging 0.25–0.61 μg) are well below pharmacologically active doses of NBOMes (usually 0.5–1 mg [7]), we cannot exclude the possibility that metabolic route and excretory rate differ in these settings.

To further explore the biodistribution of 5-HT_2A/2C_ receptors and metabolism, whole-body scans after pre-treatment with blocking agents such as Ketanserin (5-HT_2A_ receptor antagonist) or CYP inhibitors could be performed, and CYP isoform profiling of participants could be correlated.

## Conclusion

The 5-HT_2A_ receptor agonist PET radioligand ^11^C-Cimbi-36 has a favorable radiation dosimetry profile with an effective dose of 5.5 μSv/MBq, which allows for injection of 1.8 GBq per study in healthy volunteers, thus making ^11^C-Cimbi-36 eligible for studies involving a series of PET scans in a single subject.

The biodistribution reflects the rapid metabolism and glucuronidation of ^11^C-Cimbi-36 in the liver and subsequent excretion of this metabolite through the kidneys and gallbladder, but also shows considerable variability, that might play into the toxicity seen for the unlabeled version, 25B-NBOMe, when used as a recreational drug. Surprisingly, uptake throughout the scan was seen in periclavicular brown adipose tissue for four subjects, quite possibly representing specific 5-HT_2A/2C_ receptor binding.

## Additional file

Additional file 1: TIAC values. (XLSX 30 kb)

## Data Availability

Authors can confirm that all relevant data are included in the article and/or its supplementary information files.

## Abbreviations

5-HT_2A_: Serotonin 2A receptor
AUC: Area under the curve
BAT: Brown adipose tissue
CYP: Cytochrome P-450 enzyme
PET: Positron emission tomography
SUV: Standard uptake value
TAC: Time-activity curve
TIAC: Time-integrated activity coefficient
UGT: Glucuronosyltransferases
VOI: Volume of interest
WB: Whole blood
